# Supplementary material for: Identification of Freezing-Responsive microRNAs and Their Targets in Chinese Jujube by Small RNA and Degradome Sequencing
Source: Int J Mol Sci. 2026 Sep 4;27(17):7912. doi: 10.3390/ijms27177912 (PMC13566890; doi:10.3390/ijms27177912)
Supplement: Supplementary file 1 [file ijms-27-07912-s001.zip › Supplementary Material S2/novel_miR_154.pdf]

Provisional ID	:	novel_miR_154
Score total	:	0.7
Score for star read(s)	:	-1.3
Score for read counts	:	0
Score for mfe	:	0.4
Score for randfold	:	1.6
Score for cons. seed	:	
Total read count	:	387
Mature read count	:	387
Loop read count	:	0
Star read count	:	0

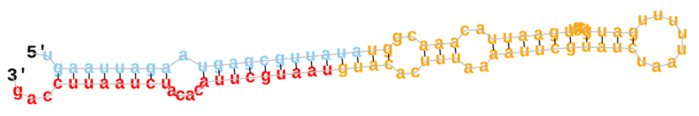

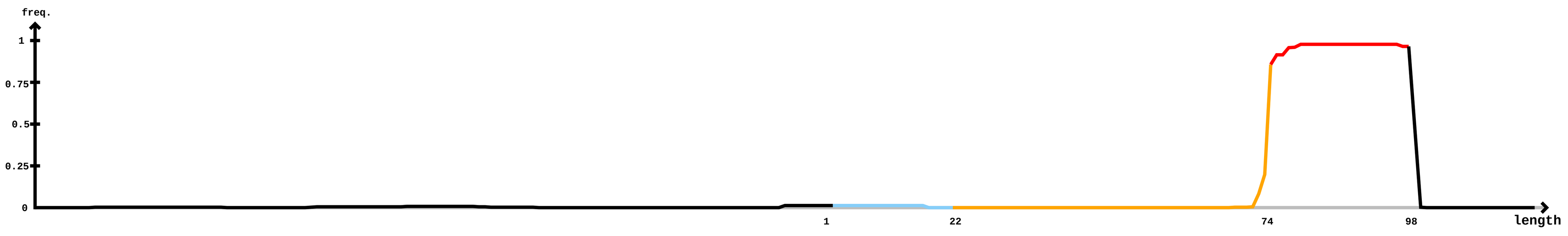

Star Mature

5'- gguugcuacaauuuugucucccagcucuggcaugcugcccuuguguugagcauguugauuguaguaguuauggucguccacaugccaugauaugucauuuaugccauugaguacgauuaguuuauugauguugaauuagaaugagcguuauauggcaaacauuaaguugcuguaguuuuuaaucuaugcuuaaaauuucacauguaaugcuuacacaucuaauuccaggauggcaucuguaauaucaaag -3' exp

..((((((((((.((((((...((...((((...))))...))...))).)))..)))))))))))...(((.(((....))))))((((((..((...((((((((.....((((......)))).....((((((((.((((((((((((..(((..((((((....((((.......))))))))))..)))..))))))))))))....))))))))...)))))))).))..))))))...

read	reads	mm	sample
caauuuugucucccagcucugg	1	0	LT-3
guugagcauguugauuguaguaguuaug	1	0	LT-2
uugagcauguugauuguaguaguuauggu	1	0	LT-3
guaguaguuauggucguccaca	1	0	LT-1
auugauguugaauuagaaugagcg	3	0	JCK-3
auugauguugaauuagaaugagcg	2	0	JCK-2
cacauguaaugcuuacacaucuaauuccag	1	0	JCK-1
Cuguaaugcuuacacaucuaauuccag	1	1	LCK-3
uguaaugcuuacacaucuaauuccag	2	0	LT-3
uguaaugcuuacacaucuaauuccag	2	0	LT-2
uguaaugcuuacacaucuaauuccag	1	0	JCK-1
uguaaugcuuacacaucuaauuccag	18	0	LCK-3
uguaaugcuuacacaucuaauuccag	1	0	LT-1
uguaaugcuuacacaucuaauuccag	2	0	JCK-2
uguaaugcuuacacaucuaauuccag	1	0	JCK-3
uguaaugcuuacacaucuaauuccagA	4	1	LT-2
guaaugcuuacacaucuaauuccag	3	0	LT-3
guaaugcuuacacaucuaauuccag	3	0	JCK-3
guaaugcuuacacaucuaauuccag	11	0	JCK-2
guaaugcuuacacaucuaauuccag	3	0	JCK-1
guaaugcuuacacaucuaauuccag	3	0	LT-2
guaaugcuuacacaucuaauNccag	1	1	JCK-1
guaaugcuuacacaucuaauuccag	14	0	LCK-3
guaaugcuuacacaucuaauuccag	7	0	LT-1
guaaugcuuacacaucuaauuccagga	1	0	LT-3
uaaugcuuacacaucuaauuccag	1	0	JT-2
uaaugcuuacacaucuaauuccag	35	0	LT-2
uaaugcuuacacaucuaCuuccag	1	1	LCK-3
uaaugcuuacacaucuaauuccag	35	0	JCK-2
uaaugcuuacacaucuaauuccGg	1	1	JCK-2
uaaugcuuacacaGcuaauuccag	1	1	JT-1
uaaugcuuacacaucuaauuccag	16	0	JCK-3
uaaugcuuacacaucuaauuccGg	1	1	JCK-1
uaaugcCuacacaucuaauuccag	1	1	JT-1

Star Mature

gguugcuacaauuuugucucccagcucuggcaugcugcccuuguguugagcauguugauuguaguaguuauggucguccacaugccaugauaugucauuuaugccauugaguacgauuaguuuauugauguugaauuagaaugagcguuauauggcaaacauuaagguugcuguaguuuuuaaucuaugcuuaaaauuucacauguaaugcuuacacaucuaauuccaggauggcaucuguaauaucaaag

uaaugcuuacacaucuaauuccag	1	0	JT-3
uaaugcuuacCcaucuaauuccag	1	1	LT-1
uaCugcuuacacaucuaauuccag	1	1	LT-2
uaaugcuuacacaucuaauuccag	55	0	LCK-3
uaaugcuuacacaucuaauuccag	23	0	LT-1
uaaugcuuacacaucuaauuccag	20	0	LT-3
uaaugcuuacacaucuaauuccag	40	0	JT-1
uaaugcuuacacaucuaauuccag	23	0	JCK-1
uaaugcuuacacaucuaauuccagA	1	1	LT-1
uaaugcuuacacaucuaauuccagA	5	1	LCK-3
aaugcuuacacaucuaauucc	2	0	JCK-3
aaugcuuacacaucuaauucc	3	0	JCK-1
aaugcuuacacaucuaauuccag	2	0	JCK-2
aaugcuuacacaCcuaauuccag	1	1	LCK-3
aaugcuuacacaucuaauuccag	4	0	LT-1
aaugcuuacacaucuaauuccag	5	0	LCK-3
aaugcuuacacaucuaauuccag	3	0	LT-2
aaugcuuacacaucuaauuccag	3	0	LT-3
ugcuuacacaucuaauuccag	4	0	LT-2
ugcuuacaGaucuaauuccag	1	1	LT-2
ugcuuacacaucuaauuccag	3	0	JT-3
ugcuuacacaucuaauuccag	6	0	JT-2
ugcuuacacaucuaauuccag	2	0	LT-1
ugcuuacacaucuaauuccagA	1	1	LT-2
gcuuacacaucuaauuccag	1	0	LT-1
cuuacacaucuaauuccag	6	0	LCK-3
cuuacacaucuaauuccag	1	0	JCK-3
